# GPER limits adverse changes to Ca^2+^ signalling and arrhythmogenic activity in ovariectomised guinea pig cardiomyocytes

**DOI:** 10.3389/fphys.2022.1023755

**Published:** 2022-11-10

**Authors:** Alice J. Francis, Jahn M. Firth, Jose L. Sanchez-Alonso, Julia Gorelik, Kenneth T. MacLeod

**Affiliations:** National Heart and Lung Institute, Imperial College, Hammersmith Hospital, London, United Kingdom

**Keywords:** oestrogen, anti-arrhythmia, cardiac excitation contraction coupling, cardiacelectrophysiology, GPER activation, GPER agonist G-1, ovariectomised, menopause

## Abstract

**Background:** The increased risk of post-menopausal women developing abnormalities of heart function emphasises the requirement to understand the effect of declining oestrogen levels on cardiac electrophysiology and structure, and investigate possible therapeutic targets, namely the G protein-coupled oestrogen receptor 1 (GPER).

**Methods:** Female guinea pigs underwent sham or ovariectomy (OVx) surgeries. Cardiomyocytes were isolated 150-days post-operatively. Membrane structure was assessed using di-8-ANEPPs staining and scanning ion conductance microscopy. Imunnohistochemistry (IHC) determined the localisation of oestrogen receptors. The effect of GPER activation on excitation-contraction coupling mechanisms were assessed using electrophysiological and fluorescence techniques. Downstream signalling proteins were investigated by western blot.

**Results:** IHC staining confirmed the presence of nuclear oestrogen receptors and GPER, the latter prominently localised to the peri-nuclear region and having a clear striated pattern elsewhere in the cells. Following OVx, GPER expression increased and its activation reduced Ca^2+^ transient amplitude (by 40%) and sarcomere shortening (by 32%). In these cells, GPER activation reduced abnormal spontaneous Ca^2+^ activity, shortened action potential duration and limited drug-induced early after-depolarisation formation.

**Conclusion:** In an animal species with comparable steroidogenesis and cardiac physiology to humans, we show the expression and localisation of all three oestrogen receptors in cardiac myocytes. We found that following oestrogen withdrawal, GPER expression increased and its activation limited arrhythmogenic behaviours in this low oestrogen state, indicating a potential cardioprotective role of this receptor in post-menopausal women.

## 1 Introduction

Menopause is associated with changes in cardiovascular health, with post-menopausal women experiencing more cardiac risk events (Amin et al., 2020) and vulnerability to cardiovascular disease (Maslov et al., 2019) and syndromes such as Takotsubo cardiomyopathy (Shufelt et al., 2018), than pre-menopausal women. These changes have been linked with declining oestrogen levels at menopause. Women are now expected to live a third of their life in a post-menopausal state (Debén et al., 2015), so enhancing our understanding of the effect of hormone changes and in particular, oestrogen signalling in the heart is fundamental to developing effective therapies and lessening the burden of disease.

We previously reported that the long-term absence of ovarian hormones in the guinea pig resulted in detrimental changes to cardiomyocyte Ca^2+^ handling mechanisms that were pro-arrhythmic and led to dysynchronous excitation-contraction (EC) coupling (Yang et al., 2017). These changes to Ca^2+^ regulation and the formation of early and delayed afterdepolarisations (EAD/DADs) were alleviated by treatment with 17β-oestradiol, strengthening the hypothesis that circulating oestrogens have direct actions on the heart. These actions are likely mediated by cardiac oestrogen receptors; oestrogen receptor alpha (ERα), oestrogen receptor beta (ERβ) and G-protein coupled oestrogen receptor 1 (GPER).

There is increasing interest in the role of GPER in the heart. It has been shown that cardiac-specific GPER knockout mice have upregulated hypertrophic genes and adverse remodelling leading to LV dysfunction (Wang et al., 2017). Additionally, GPER activation has been found to improve myocardial relaxation and attenuate hypertrophy in hypertensive rats (di Mattia et al., 2020; Jessup et al., 2010), and be cardioprotective in both male and female rat hearts subjected to ischaemia followed by reperfusion (Deschamps and Murphy, 2009). More recently, GPER agonism has been shown to regulate the adrenergic response in cardiomyocytes through abolishing isoprenaline-mediated increases in Ca^2+^ current, ectopic contractions (Whitcomb et al., 2020) and increasing cAMP levels (Machuki et al., 2019). These observations suggest the receptor plays an important role in modulating cardiac physiology, contractility and remodelling.

GPER binds oestrogen (Luo and Liu, 2020) so we wished to determine if it could be responsible for mediating the changes in Ca^2+^ handing we observed in earlier work following withdrawal and replacement of oestrogen (Yang et al., 2017). We wished to test if the selective activation of GPER using G-1 could improve disrupted Ca^2+^ signalling and limit pro-arrhythmic activity in cardiac myocytes isolated from the hearts of sham and ovariectomised guinea pigs. G-1 has already shown promising effects in improving outcomes in diabetes and obese patients and is being developed as a prototype agent for treatment for these disorders (Sharma and Prossnitz, 2021). The testing of the effects of G-1 on these cells will help determine the potential cardioprotective effect of targeting cardiac myocyte GPER in response to oestrogen withdrawal.

We use our established model of oestrogen withdrawal produced by ovariectomy (Yang et al., 2017; Firth et al., 2020) and find that GPER activation may mitigate some of the abnormal Ca^2+^ regulation and alterations to EC coupling reported in our ovariectomised guinea pig cardiomyocytes (Yang et al., 2017).

## 2 Materials and methods

### 2.1 Animal model

#### 2.1.1 Ethical approval

All studies were carried out with the approval of the Animal Welfare and Ethical Committee of Imperial College and the Home Office, United Kingdom and are in accordance with the United Kingdom Home Office Guidance on the Operation of the Animals (Scientific Procedures) Act 1986, which conforms to the Guide for the Care and Use of Laboratory Animals published by the US National Institute of Health under assurance number A5634-01.

#### 2.1.2 Surgical procedures

Female Dunkin-Hartley guinea pigs (Marshall BioResources) were randomly allocated to experimental groups and housed at 21°C on a 12 h light-dark cycle with standard feed and water *ad libitum*. Anaesthesia was induced and maintained with combined 2–4% isoflurane/95% oxygen inhalation using a rodent mask (VetTech Solutions Ltd.). Pre-operative medication was administered by subcutaneous injections: 0.05 mg kg^−1^ atropine sulphate (Atrocare, Animalcare Ltd.), 5 mg kg^−1^ enrofloxacin (Baytril, Bayer), 5 mg kg^−1^ carprofen (Rimadyl, Pfizer) and 0.05 mg kg^−1^ buprenorphine (Vetsergic, Alstoe). In addition, 4 mg kg^−1^. h^-1^ saline 0.9% was administered for intra-operative hydration. The local anaesthetic bupivacaine (2 mg kg^−1^ (Macaine Polyamp, AstraZeneca)) was administered subcutaneously around the incision sites. Post-operative analgesic carprofen was given orally for 2 days (5 mg kg^−1^).

Bilateral flank ovariectomy (OVx) or sham gonad-intact surgeries were conducted on anaesthetised animals weighing 350–450 g as described previously (Yang et al., 2017). Animals were housed for 150-days post-surgery to establish a consistent low oestrogen environment (OVx), in parallel with a timed-control group (sham).

#### 2.1.3 Serum hormone concentrations

At 150-days post-opertatively, blood samples were taken and collected in a serum separator tube (Becton Dickinson). After 2 h, they were centrifuged at 1,300 *g* for 15 min at 4°C. The supernatant (serum) was stored at −80°C. Sandwich enzyme-linked immunosorbent assays (ELISA) used according to manufacturer’s instructions (all from BioSource) determined serum levels of 17β-oestradiol. The mean absorbance density was recorded using a spectrometer set at 450 nm excitation. All samples were run in triplicate and blank corrected. The standard curves were produced and interpolated where necessary using GraphPad Prism 9.0. An online resource of mycurvefit.com was used to interpolate mean absorbance density of each sample to quantify the concentration, based on the standard curve.

### 2.2 Cardiomyocyte isolation

The heart and lungs were explanted from anaesthetised guinea pigs in a non-recovery regulated procedure under isoflurane/oxygen inhalation and placed in ice-cold Krebs-Henseleit (KH) solution (NaCl 119 mm, KCl 4.7 mm, MgSO_4_ 0.94 mm, CaCl_2_ 1 mm, KH_2_PO_4_ 1.2 mm, NaHCO_3_ 25 mm, glucose 11.5 mm, pH 7.4) containing heparin (500 U/ml). The heart was carefully trimmed, weighed, the aorta cannulated (12G cannula) and attached to a Langendorff retrograde perfusion system maintained at 37°C and set to 6–8 ml/min/g. First, the heart was perfused with KH for 2 min then perfusion was switched to a low Ca^2+^ solution (NaCl 120 mm, KCl 5.4 mm, CaCl_2_ 0.015 mm, MgSO_4_ 5 mm, C_3_H_3_NaO_3_ 5 mm, glucose 20 mm, taurine 20 mm, HEPES 10 mm, NTA 5 mm, pH 6.96) for 4 min. Finally, the heart was perfused with enzyme solution (NaCl 120 mm, KCl 5.4 mm, CaCl_2_ 0.2 mm, MgSO_4_ 5 mm, C_3_H_3_NaO_3_ 5 mm, glucose 20 mm, taurine 20 mm, HEPES 10 mm, pH 7.4) containing Liberase TL enzyme (100 μg/ml) blend (Roche Diagnostics) for tissue digestion and dissociation. Digested LV tissue was sectioned and mechanically dissociated in ES before filtering the tissue through a fine mesh and centrifuging (400 rpm for 1 min) to isolate single cardiomyocytes. In addition to the heart and LV, the lungs and uterus were weighed and normalised to body weight for each animal.

### 2.3 Experimental protocols

Cardiomoycytes from sham or OVx were re-suspended and stored in a working solution of 1:1 enzyme solution (without active enzyme) and Dulbecco’s Modified Eagle medium (Thermo Fisher Scientific) at room temperature (RT) for up to 6–8 h. Cardiomyocytes were divided into the control (ctrl) condition or pre-conditioned with the GPER agonist G-1 (1 µm (Tocris Bioscience)) in working solution for a minimum of 2 h before use. In the G-1 group, 1 µm G-1 was continuously present in superfusate solutions. This concentration of G-1 was determined by dose-dependency experiments, shown in Supplementary Figure 2. The dose-dependency titration was stopped at 10 µm as it is suggested that G-1 may interact with microtubules at higher doses (Holm et al., 2012; Wang et al., 2013) and with oestrogen response elements in the promoter regions of ERα and ERβ genes at concentrations >10 µm (Bologa et al., 2006; Dennis et al., 2011).

### 2.4 Immunohistochemistry (IHC) and western blotting

#### 2.4.1 Oestrogen receptor localisation

Cardiomyocytes were fixed with 4% paraformaldehyde [in phosphate-buffered saline (PBS)] and permeabilised with 0.01% Triton (10 min), prior to 30 min incubation with 10% bovine serum albumin (BSA). For t-tubule staining, wheat germ agglutinin (WGA, Invitrogen) (1:20 with PBS) was added to cells prior to blocking. Cells were incubated overnight at 4°C with anti-ERα (sc-543, Santa Cruz Biotechnology), anti-ERβ (sc-390243, Santa Cruz Biotechnology), anti-GPER (*ab39742,* Abcam) primary antibody diluted in BSA (all 1:150). The secondary antibody diluted in BSA (1:400) (goat anti-rabbit Alexa-488 (A11008, Invitrogen) or donkey anti-mouse Alexa-488 (A21202, Invitrogen) was applied for 3 h at RT. Two (2 min) washes with PBS were completed after antibody incubations. Lastly, Vectashield with Dapi (Vector Laboratories) was added. A Zeiss LSM780 confocal microscope (63x oil) was used to acquire z-stack images with standardised settings.

In FIJI (Schindelin et al., 2012), image threshold was set and the fluorescence intensity of the protein of interest measured within a 21 µm × 11 µm region of interest (ROI 1). ROI one was placed in the centre of the cell (avoiding the nucleic and peri-nucleic regions) and a second ROI with the same dimensions (ROI 2) was placed at the edge of the cytosolic region. The ROI measurements were averaged for each of the five z-stacks, providing a mean fluorescence value for each cell. Analyses were conducted with the experimenter blinded to the groups.

#### 2.4.2 Western blotting

Cardiomyocytes were lysed and proteins quantified using Pierce™ BCA Protein Quantification Kits. Proteins (30 µg) were loaded onto Bio-Rad Criterion™ 10% pre-cast mini gels and separated by electrophoresis using XT MOPS running buffer and a Bio-Rad Criterion™ Vertical Electrophoresis Cell. Proteins were blotted onto a nitrocellulose membrane using a Criterion™ Blotter (1 h at 100 V at 4°C). Membranes were blocked for 1 h at RT before being divided and probed with anti-GPER (1:500), anti-ERα (1:5,000), anti-ERβ (1:1,000), and for housekeeping anti-GAPDH (1:20,000, sc-47724, Santa Cruz Biotechnology) overnight at 4°C. Next, membranes were washed with Tris-buffered saline with 0.1% Tween^®^ 20 before the application of secondary antibody (1:2000, anti-rabbit IgG-HRP, 7074S, Cell Signalling Technology or anti-mouse IgG-HRP, ab205719, Abcam) for 1 h at RT. The membranes were washed and visualised using Bio-Rad Clarity Western ECL Substrate and captured using the Bio-Rad ChemiDoc MP.

#### 2.4.3 Electrophysiological experiments

Electrophysiological recordings were acquired using sharp micro-pipettes filled with high K^+^ solution (KCl 2 M, EGTA 0.1 M, HEPES 5 mm, pH 7.2) (15–20 MΩ) and connected *via* an Axon HS-2A x0.1LU headstage to an Axoclamp2B switch-clamp (Molecular Devices). The signals were processed through a Digidata 1440A and ClampEx 10.7 software (Molecular Devices). Cells were visualised using an inverted Nikon microscope (40x) and superfused with appropriate solutions at 37°C. All current measurements were normalised to current density, quantified by measuring the capacitance of each cell. Clampfit 10.7 (Molecular Devices) was used for all analyses.

Action potentials (APs) were generated using 2.5–3 nA depolarising current pulses applied for up to 3 ms at 0.5 Hz and analysed from a signal averaged trace produced from 25 APs. To record L-type Ca^2+^ channel current (*I*_Ca,L_) and its current-voltage (I-V) relationship, cells were voltage clamped at −40 mV using the AxoClamp 2B amplifier in switch clamp mode (gain: 0.5–1.5 nA/mV and switching rate 2–4 kHz) and a train of 200 ms depolarising clamp steps (from −45 mV to +55 mV in 5 mV increments) applied at 0.5 Hz (see Figure 3A). The clamp protocols were controlled using pClamp 10 (Molecular Devices).

L-type Ca^2+^ channel (LTCC) inward Ca^2+^ current (*I*_Ca,L_) was identified as cadmium-sensitive current, subtracting the initial recorded currents from those recorded in the presence of 200 µm CdCl_2_. The voltage-dependence of activation and inactivation were fitted with the Boltzman equation: {G = G_max_ × [1 + exp (V1/2−V)/κ] −1} where G is the conductance at various test potentials, calculated from the peak current according to: G = I/(V-E_rev_). E_rev_ is the reversal potential obtained by extrapolating the linear part of the I-V curve to its intersection with the voltage axis. G_max_ is maximum conductance; V1/2 and κ are half-activation voltage and the slope factor. The activation current is presented as G/G_max_ and inactivation as I/I_max_.

To increase the occurrence of early afterdepolarisations (EADs) cells were superfused with either: 1) 50 nm isoprenaline for 30 s or 2) 1 µm dofetilide for 5 min. These drugs were superfused in the presence or absence of G-1 and membrane potential recorded during 0.5 Hz stimulation.

#### 2.4.4 Sarcoplasmic reticulum (SR) Ca^2+^ content

Ca^2+^ uptake into the SR is facilitated *via* the sarcoplasmic reticulum Ca^2+^ ATPase (SERCA) pump. SR Ca^2+^ content was measured by rapid application of caffeine to voltage-clamped cells held at −80 mV following a 40 s 1 Hz stimulation train. Caffeine activates ryanodine receptors (RyR) causing complete efflux of Ca^2+^ from the SR. The released Ca^2+^ is then extruded from the cell *via* Na^2+^−Ca^2+^ exchanger (NCX) in forward mode and the net inward flow of positive charge induced by the efflux of Ca^2+^ is integrated and provides an estimate of the Ca^2+^ content of the SR. This is based on the assumptions that all the Ca^2+^ was released during the caffeine application and the stoichiometry of the exchange is 3 Na^+^:1 Ca^2+^.

### 2.5 Fluoresence Ca^2+^ measurements

#### 2.5.1 Fluoresence Ca^2+^ dye loading

##### 2.5.1.1 Ratiometric dyes: Fura-4, AM

For Ca^2+^ transient (CaT) recordings using the CytoCypher, cells plated on MatTek dishes were incubated with 2 ml working solution containing 1 µm Fura-4, AM (Invitrogen, Life Technologies Ltd.) for 25 min at RT. The solution was replaced with fresh working solution for a 20 min de-esterification period. NT (±G-1) bathed the cells during the recordings.

##### 2.5.1.2 Non-ratiometric dyes: Fluo-4, AM

For SR fractional release measurements and Ca^2+^ sparks, myocytes were loaded with 10 µm Fluo-4, AM (25 min at RT). Loaded cells were collected by low speed centrifugation and the solution replaced with fresh working solution (±G-1) for 30 min at RT to allow for the de-esterification of the Ca^2+^ indicator.

### 2.6 Fluorescence Ca^2+^ experiments

#### 2.6.1 CaT measurements

General CaT and sarcomere length (SL) shortening data were acquired simultaneously using the high-throughput CytoCypher™ system with a static high-resolution camera and a microscope supplemented by a dual-excitation LED light source and photomultiplier detector. The system autonomously selects cells to record, avoiding operator bias. Cells were paced at 0.5 Hz and transients recorded for 10 s at 37°C. Ca^2+^ and contractility data were analysed using CytoSolver™ as described in previous work (Wright et al., 2020). CaT amplitude was expressed as the Fura-4 ratio (360/380 nm).

#### 2.6.2 SR release and Ca^2+^ efflux mechanisms

Rapid application of 10 mm caffeine to Fluo-4-loaded cells induced an increase in Ca^2+^ (CaffT) described previously (Yang et al., 2017). The decay phase of the CaffT was fitted with a single-exponential function to measure the time and rate constant of NCX and slow transporters. The rate of SERCA-dependent uptake was measured by subtracting the rate constant of decay of the CaffT from the steady-state CaT and with the assumption that during caffeince application SERCA does not contribute to the decline of the CaffT.

#### 2.6.3 Ca^2+^ spark and wave assessment

Fluo-4 loaded cells within an open diamond RC24N superfusion chamber (Warner Inc.) were visualised using a ×40 oil-immersion objective, attached to an inverted Nikon Eclipse TE-300 microscope complete with a Bio-Rad Radiance 2000 confocal attachment. The argon laser was set at 14.5% power and experiments performed at 37°C in low-light conditions. Recordings were acquired using LaserSharp1000 software and line-scan mode (15,000 lines at 750 lines/s). Myocytes were first paced at steady-state (0.5 Hz) then 1 Hz for 20 s. Field stimulation was stopped and cells recorded for 20 s. Analysis was conducted using SparkMaster or Wave Data plugins from ImageJ. Spark detection criteria was set at 4.2 times the standard deviation above the mean background value. The average amplitude of the sparks arising in each cell was calculated. Wave frequency was calculated as the number of waves divided by the total recording time (20 s) and wave-free survival time was measured from the time of the decay of the last Ca^2+^ transient to the start of the first Ca^2+^ wave.

### 2.7 Statistical analyses

Statistical analyses were performed and figures produced using GraphPad Prism 9.0 (GraphPad Software). Data are presented as mean ± standard deviation (SD) or mean ± 95% confidence interval (CI) with n being the number of cells and N the number of hearts (n/N = cells/hearts). *p* values are designated with asterisks: *p < 0.05, **p < 0.01, ***p < 0.001. The statistical test used is detailed in each figure legend. Hierachial (nested) approaches were used where applicable.

## 3 Results

### 3.1 Confirmation of model as ovariectomised animals had reduced circulating oestrogens

There was no difference in body weight between sham and OVx animals. The OVx model was confirmed through the significant (73%) reduction in uterine weight in animals that underwent OVx surgery. It is noteable that the withdrawal of oestrogen also induced reduction in lung and LV weight to body weight ratio (Table 1).

**TABLE 1:** Weight ratios and blood serum concentrations of 17β-oestradiol from sham and OVx.

Experimental model	N of animals	Body weight (g)	Weight ratio to body weight (g/kg)				Serum concentration 17β-oestradiol (pg/ml)
			Lung	Heart	Left ventricle	Uterus	
Sham	12	860.7 ± 76.9	4.5 ± 0.4	2.8 ± 0.2	1.7 ± 0.2	1.5 ± 0.5	1.6 ± 0.5
OVx	11	903.3 ± 73.6	4.1 ± 0.3 **p = 0.008	2.6 ± 0.2	1.5 ± 0.2 *p = 0.03	0.4 ± 0.1 ***	0.8 ± 0.4 ***p < 0.0001

The effect of ovariectomy surgery was assessed by measuring the levels of 17β-oestradiol in the blood serum. 17β-oestradiol concentration reduced by 44% following OVx compared with sham females (Table 1). It is noteworthy that the sham animals were sacrificed at random times during their menstrual cycle.

### 3.2 Localisation of oestrogen receptors and increased expression of GPER in OVx

**FIGURE 1:**
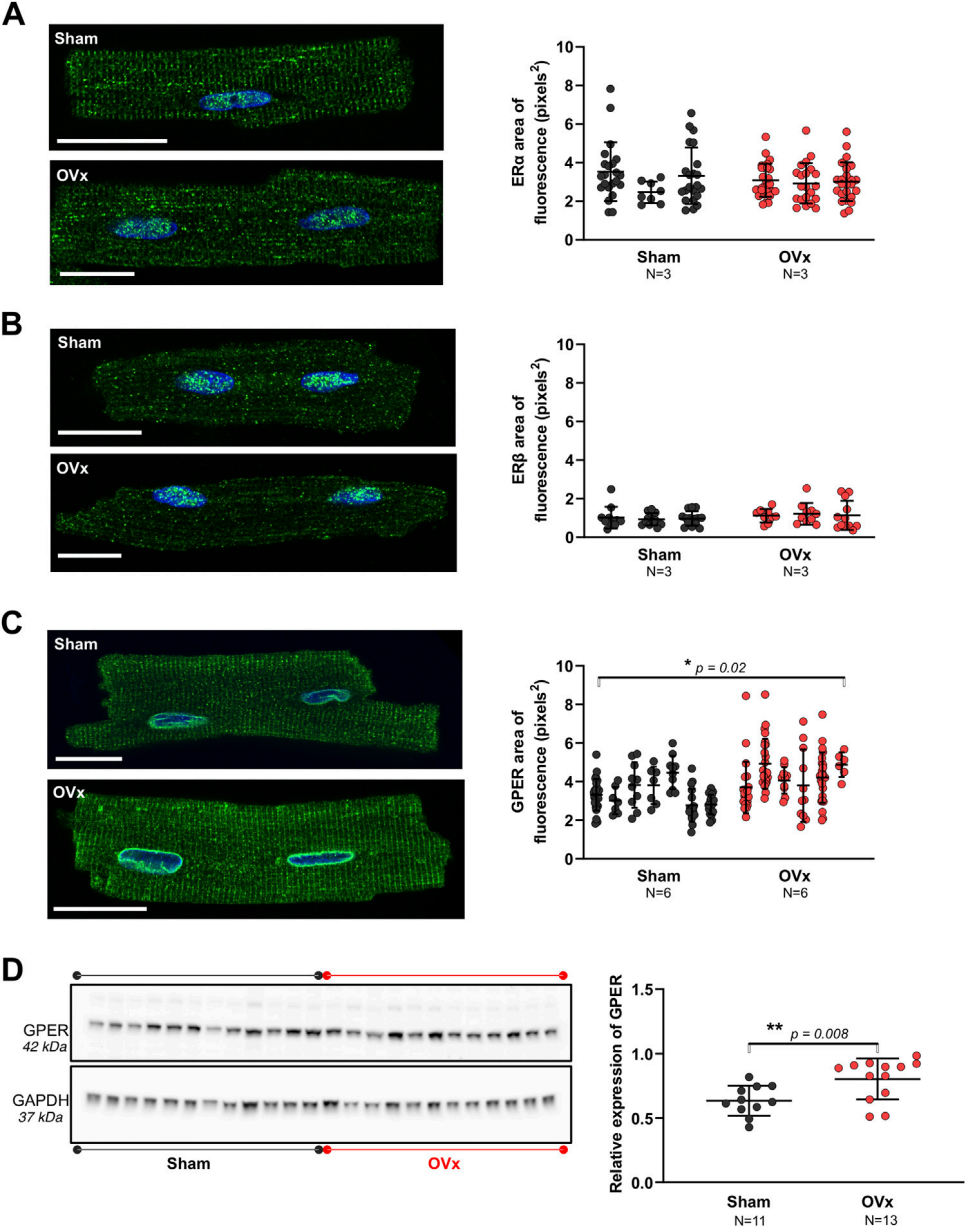
The presence of oestrogen receptors in sham and OVx cardiomyocytes. **(A)** ERα localised to the nuclei and showed some cytosolic staining, with no change in fluorescence between sham and OVx. Mean ± SD. Student’s t-test. Sham n/N = 53/3; OVx n/N = 74/3. **(B)** ERβ was predominantly found in the nucleus, with no difference in OVx. Mean ± SD. Student’s t-test. Sham n/N = 40/3; OVx n/N = 31/3. **(C)** GPER was highly localised to the peri-nuclear ER and showed striated staining within the cytosol, which was higher in OVx compared with sham. Mean ± SD. Student’s t-test. *p < 0.05, value as indicated. Sham n/N = 96/6; OVx n/N = 101/6. White scale bars = 20 µm. **(D)** GPER protein expression was higher in OVx compared with sham. Each data point in the graph represents the average protein ratio from each heart from three repeat blots. Mean ± SD. Student’s t-test. **p < 0.01, value as indicated. Sham *N* = 11; OVx *N* = 12–13. Protein loading controls were run on the same blot, but different exposure times are presented for GPER (25 min) and GAPDH (2 min).

ERα showed dense fluorescence within the nuclei and some cytosolic staining (Figure 1A). The cytosolic stain of ERβ was sparse, with the protein expression mostly confined to the nuclei (Figure 1B). GPER was expressed throughout the cells but dense staining occurred at the internal membrane surrounding the nucleus, namely the peri-nuclear endoplasmic reticulum (ER) (Figure 1C). The localisation pattern of each receptor was comparable between sham and OVx cardiomyocytes. There was no change in fluorescence levels of ERα or ERβ (Figures 1A,B). Cytosolic GPER fluorescence increased by 23% in OVx compared with sham (Figure 1C). Further investigation of GPER expression in sham and OVx by western blotting, showed a 32% increase in GPER expression in low oestrogen conditions (Figure 1D).

### 3.3 Ca^2+^ transient amplitude decreased following GPER activation in OVx

**FIGURE 2:**
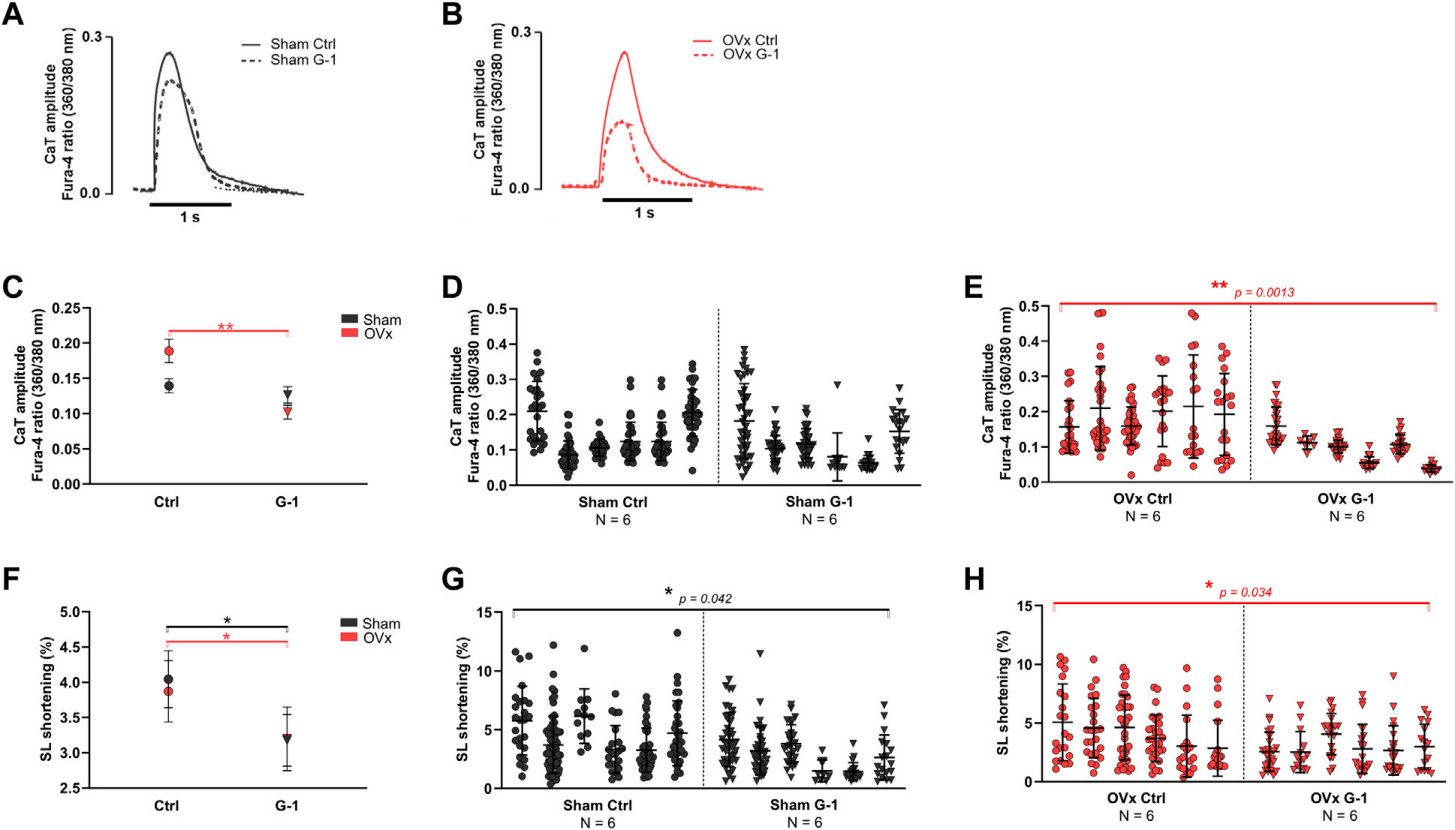
The effect of GPER activation on Ca^2+^ transient (CaT) parameters and contractility in sham and OVx, measured using high-throughput CytoCypher system. **(A)** Representative CaT traces of sham ± G-1 and **(B)** of OVx ± G-1 in Fura-4 loaded cardiomyocytes. **(C)** CaT amplitude was not changed in sham but reduced following GPER activation in OVx. Mean ± 95% CI. **(D)** Nested analyses of CaT amplitude changes following GPER activation in sham and **(E)** OVx cardiomyocytes. Mean ± SD. **p < 0.01, values as indicated. **(F)** SL shortening reduced in sham and OVx following GPER activation. Mean ± 95% CI. **(G)** Nested analyses of SL shortening changes following GPER activation in sham and **(H)** OVx cardiomyocytes. Mean ± SD. Nested t-test. *p < 0.05, Sham ctrl n/N = 207/6, sham G-1 n/N = 169/6; OVx ctrl n/N = 153/6, OVx G-1 n/N = 115/6.

Ca^2+^ transient amplitudes were not altered in sham (Figure 2D) but reduced by 46% in OVx following the addition of G-1 (Figure 2E). GPER activation had no effect on transient kinetics (not shown) but it decreased sarcomere shortening in both sham (by 15%, Figure 2G) and OVx (by 32%, Figure 2H).

A comprehensive assessment of the effects of GPER activation on SR Ca^2+^ release and re-uptake during systole is provided in Supplementary Figure S3 whereby SR Ca^2+^ content increased by 35% in OVx compared with sham but was not altered following GPER activation in sham or OVx.

### 3.4 LTCC activity increased in OVx but was not altered following GPER activation

**FIGURE 3:**
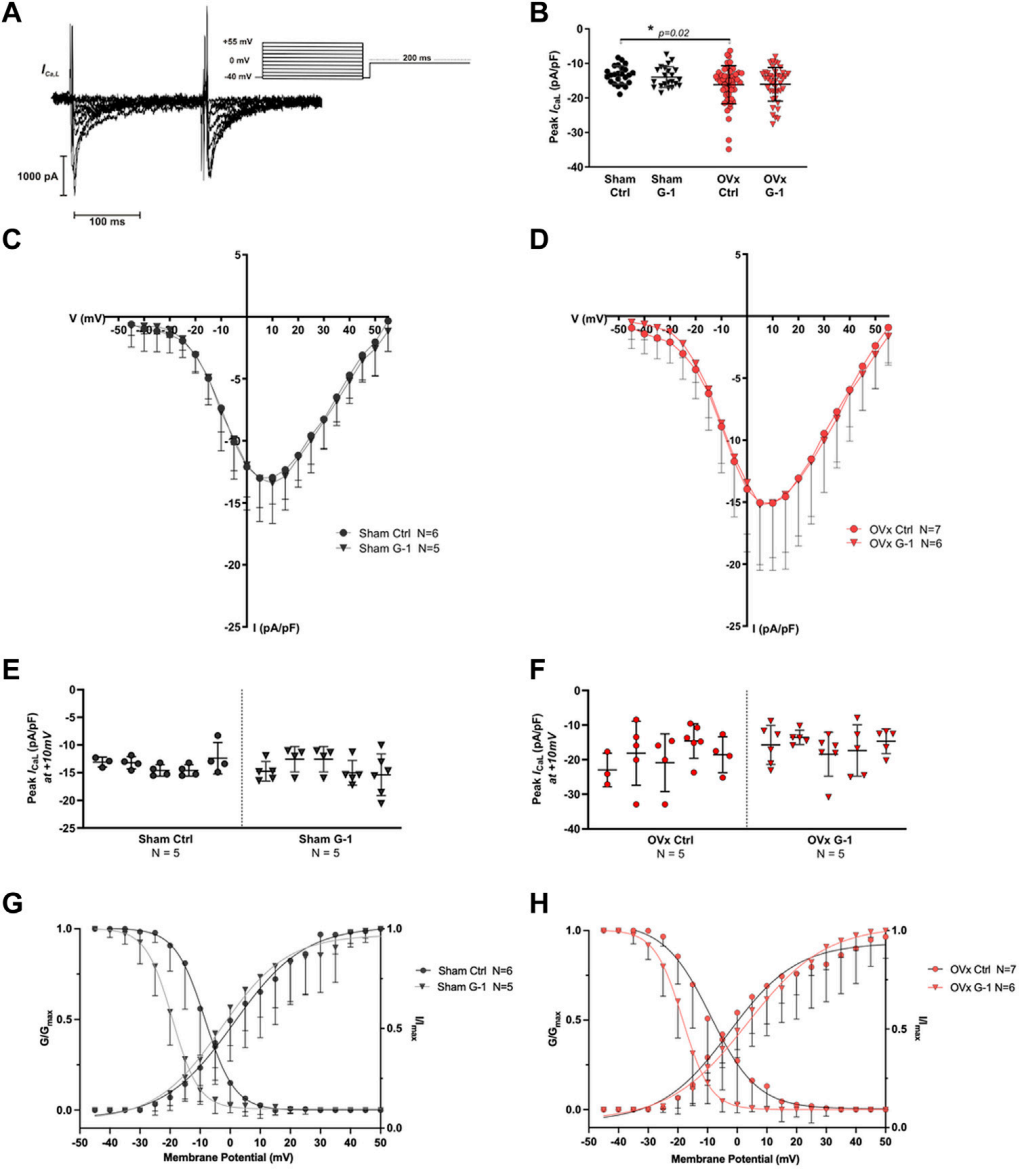
The effect of GPER activation on L-type Ca^2+^ channel (LTCC) current (*I*_Ca,L_). **(A)** Voltage-clamp step protocol to measure the current-voltage (I–V) relationship of the LTCC and representative cadmium-sensitive current trace. **(B)** The peak *I*_Ca,L_ at +10 mV was higher in OVx; however, peak *I*_Ca,L_ was unchanged following GPER activation. **(C)** I-V relationship of sham ± G-1 and **(D)** of OVx ± G-1. Mean ± SD. One-way ANOVA. *p < 0.05, value as indicated. Sham ctrl n/N = 23/6, sham G-1 n/N = 21/5; OVx ctrl n/N = 51/7, OVx G-1 n/N = 45/6. **(E)** Nested analyses of peak *I*_Ca,L_ in sham and **(F)** OVx cardiomyocytes in ctrl and G-1 conditions. Mean ± SD. Nested *t*-test. Sham ctrl n/N = 19/5, sham G-1 n/N = 24/5; OVx ctrl n/N = 22/5, OVx G-1 n/N = 25/5. **(G)** LTCC voltage-dependent activation (G/G_max_) and inactivation (I/I_max_) curves plotted for sham ± G-1 and **(H)** OVx ± G-1. Mean ± SD. Nested t-test. Sham ctrl n/N = 21/6, sham G-1 n/N = 20/5; OVx ctrl n/N = 28/7, OVx G-1 n/N = 26/6.

Since G-1 treatment significantly reduced CaT amplitude and sarcomere shortening in OVx, we investigated if Ca^2+^ influx through the LTCCs (*I*_Ca,L_) was modulated by GPER. A representative trace and the protocol used to record *I*_Ca,L_ is shown in Figure 3A. The peak *I*_Ca,L_ was slightly greater in OVx compared with sham but was not altered following application of G-1 (Figures 3B–F).

### 3.5 GPER activation shifted the voltage-dependance for inactivation of LTCC

The voltage-dependent activation and steady-state inactivation curves were fitted using the Boltzmann equation as described previously. There was no change in the activation curves in the presence of G-1 in sham or OVx (Figures 3G,H). The steady-state inactivation shifted to more negative potentials in sham and OVx cardiomyocytes in the presence of G-1 (Figures 3G,H), indicating that the activation of GPER may in turn cause the LTCC to inactivate at lower membrane potentials, reducing the probability of Ca^2+^ channels being open during the “window” current.

### 3.6 Spark and wave frequency decreased following GPER activation in OVx

**FIGURE 4:**
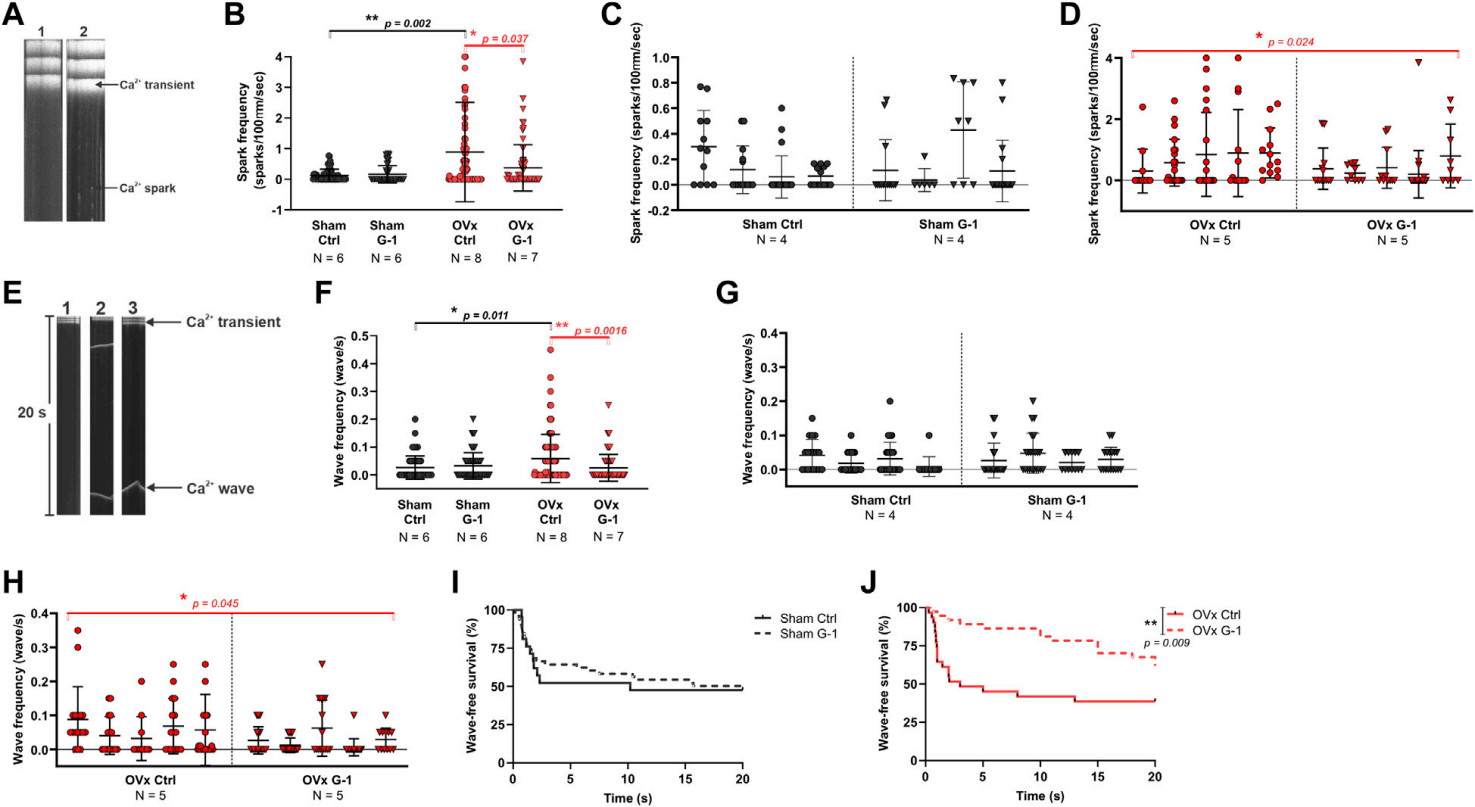
The effect of GPER modulation on spontaneous Ca^2+^ release from the SR. **(A)** Line scan images of sham and OVx cardiomyocytes loaded with Fluo-4. **(B)** Spark frequency increased in OVx compared with sham and reduced following GPER activation in OVx cells. Mean ± SD. Mann-Whitney test. *p < 0.05, **p < 0.01, values as indicated. **(C)** Nested analyses of spark frequency in sham and **(D)** OVx cardiomyocytes following GPER activation. Mean ± SD. Nested t-test. *p < 0.05, value as indicated. **(E)** Line scan of cardiomyocytes with no waves (image 1), multiple waves (image 2) and a single wave with longer wave-free survival time (image 3). **(F)** Waves frequency increased in OVx compared with sham and decreased following GPER activation in OVx. Mean ± SD. Mann-Whitney test. *p < 0.05, **p < 0.01, values as indicated. **(G)** Nested analyses of wave frequency in sham and **(H)** OVx following GPER activation. Mean ± SD. Nested t-test. *p < 0.05, value as indicated. **(I)** Wave-free survival time of sham cells was not affected by G-1 but **(J)** improved following GPER activation in OVx. Mean ± SD. Log-rank test. **p < 0.01. Sham ctrl n/N = 49/6, sham G-1 n/N = 45/6; OVx ctrl n/N = 71/8, OVx G-1 n/N = 64/7. For nested analyses: Sham ctrl n/N = 28/4, sham G-1 n/N = 30/4; OVx ctrl n/N = 49/5, OVx G-1 n/N = 46/5*.*

Line scan images of Fluo-4 labelled cardiomyocytes show the formation of Ca^2+^ sparks (Figure 4A) and waves (Figure 4E). While G-1 had no effect on spark frequency in sham (Figure 4C), it reduced Ca^2+^ sparks in OVx, decreasing the frequency by 59% (Figure 4D). Ca^2+^ waves, characterised by propagated but asynchronous increases in cytosolic Ca^2+^ during a period of electrical quiescence, are pro-arrhythmic and occurred more frequently in OVx compared with sham (Figure 4F). G-1 had no effect on wave frequency (Figure 4G) or wave-free survival time (Figure 4I) in sham animals. However, in OVx, GPER activation greatly reduced wave frequency by 56% (Figure 4H) and improved the wave-free survival time (Figure 4J).

### 3.7 GPER activation limited abnormal membrane depolarisations

**FIGURE 5:**
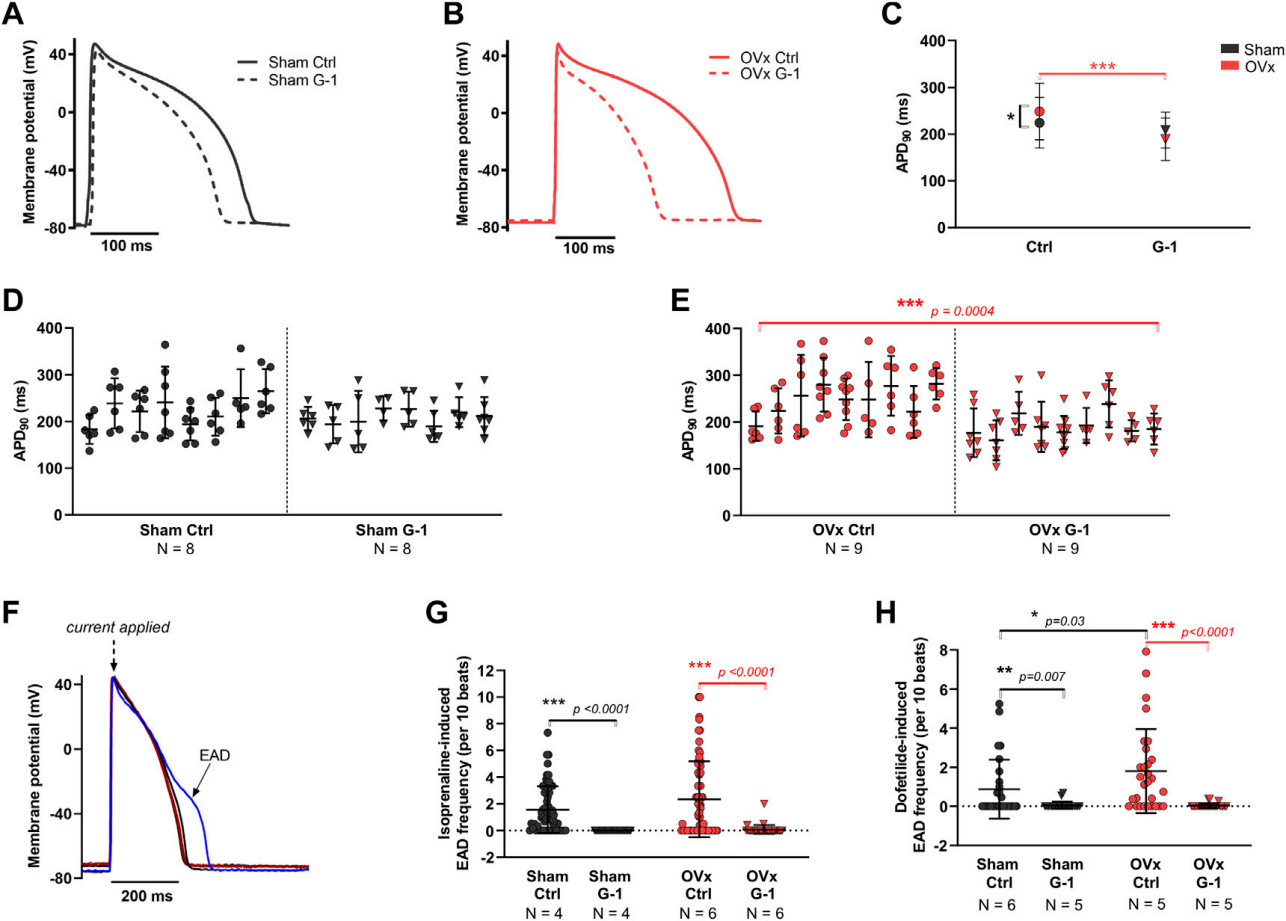
The effect of GPER on the action potential (AP) and abnormal membrane depolarisations. **(A)** Typical ventricular AP traces from sham ± G-1 and **(B)** from OVx ± G-1. **(C)** APD_90_ prolonged in OVx compared with sham and GPER activation shortened the APD_90_ in OVx cells. Mean ± 95% CI. Nested t-test. *p < 0.05, ***p < 0.001. **(D)** Nested analyses of the effect of GPER activation on APD_90_ in sham and **(E)** OVx cardiomyocytes. Mean ± SD. Nested t-test. ***p < 0.001, value as indicated. Sham ctrl n/N = 52/8, sham G-1 n/N = 46/8; OVx ctrl n/N = 59/9, OVx G-1 n/N = 57/9. **(F)** Representative traces of cardiac action potential and early after depolarisation (EAD) formations. **(G)** Isoprenaline-induced EAD frequency decreased following GPER activation in sham and OVx. Mean ± SD. Student’s t-test. ***p < 0.001, values as indicated. Sham ctrl n/N = 65/4, sham G-1 n/N = 27/4; OVx ctrl n/N = 65/6, OVx G-1 n/N = 39/6. **(H)** Dofetilide-induced EAD frequency reduced following GPER activation in sham and OVx. Mean ± SD. Mann-Whitney test. *p < 0.05, **p < 0.01, ***p < 0.001, values as indicated. Sham ctrl n/N = 26/6, sham G-1 n/N = 21/5; OVx ctrl n/N = 28/5, OVx G-1 n/N = 24/5.

Representative ventricular AP traces of sham and OVx are shown in Figure 5, respectively. GPER activation had no effect on APD in sham (Figures 5C,D). In OVx, GPER activation shortened the APD (Figures 5C,E) - the average APD_90_ shortened by 52 ms following GPER activation, 24% faster than in the OVx control condition.

The effect of GPER activation in the presence of two compounds that alter the cardiac action potential were assessed (Figure 5F). Isoprenaline, a β-adrenergic receptor (β-AR) agonist, and dofetilide, to block the delayed rectifier potassium current, *I*_Kr_ were utilised. In the presence of 50 nm isoprenaline, significantly fewer pro-arrhythmic events were recorded following GPER activation, with EAD frequency reducing by greater than 96% in sham and OVx cells in the presence of G-1 (Figure 5G). Similarly, in the presence of 1 μm dofetilide, cells incubated with G-1 had fewer EADs than control cells from both sham and OVx animals (Figure 5H).

## 4 Discussion

This is an observational study that tested if the selective activation of GPER using G-1 could improve disrupted Ca^2+^ signalling and limit pro-arrhythmic activity in cardiac myocytes isolated from the hearts of sham and ovariectomised animals. It firstly showed, using IHC, the localisation of the oestrogen receptors in guinea pig cardiomyocytes in control and low oestrogen states. From this, we identified that GPER expression was higher in OVx animals, suggesting an important role of this receptor in mediating cardiomoytcyte signalling in response to oestrogen withdrawal. This lead to further investigation into the role of GPER in cardiomyocyte Ca^2+^ signalling and it is cardioprotective potential against pro-arrhythmic activity arising in response to OVx reported previously (Yang et al., 2017). GPER is often described as being able to ‘mobilise Ca^2+^ (Brailoiu et al., 2007; Wang et al., 2012; Romano and Gorelick, 2018), however few studies have directly assessed the role of GPER in cardiomyocyte Ca^2+^ regulation for EC coupling.

### 4.1 Cardiac oestrogen receptors

This is the first study, to our knowledge, that uses IHC to show the localisation of all three oestrogen receptors in both female control cardiomyocytes and those isolated from OVx animals. The subcellular localisations of ERɑ, ERβ and GPER were distinct (Figure 1). We show ERɑ and ERβ most prominently localised to the nuclei. ERɑ had a higher level of punctate cytosolic staining than ERβ. These findings are comparable to a study in rat cardiomyocytes that showed primary expression within the nuclei but levels of ERβ were undetectable (Pugach et al., 2016). Comparable to a study in OVx rats (Mohamed and Abdel-Rahman, 2000), we show that OVx had little effect on the expression of ERɑ (Supplementary Figure S1). Although our method of protein sample preparation for western blotting was not effective in solubilising the nuclear envelope and given the sparse expression outside the nuclei, we found no change in cytosolic ERβ fluorescence in OVx and sham. These findings suggest that the withdrawal of oestrogen had little effect on cardiac ER expression. This may be dependent on cell type as ERɑ and ERβ were upregulated in OVx rat cortical and medullary renal cells (Esqueda et al., 2007) and in platelet cells from OVx pigs (Jayachandran and Miller, 2002).

The specificity of GPER antibody (*ab39742*) has been confirmed from negative control expression studies of a GPER knockdown in two gastric cancer cell lines (Xu et al., 2020) and in striatal tissue (Chen et al., 2019). GPER in cardiomyocytes highly localised to the peri-nuclear and t-tubule membranes (Figure 1), suggesting the receptor localises to the plasma membrane and intracellularly. The distribution of GPER near the t-tubules suggests its involvement in cell functions that use the complex interconnecting system and its close apposition to intracellular compartments, notably Ca^2+^ movement within the cell. Intracellular expression of GPER on the ER/nuclear membrane has been reported previously in other cell types (Revankar et al., 2005; Filardo et al., 2006) however, it is debatable whether GPER is actively signalling at these sites. A study in HEK293 cells expressing GPER found the receptor was trafficked from the plasma membrane to the ER in preparation for endocytosis, and therefore GPER may not be functional in the peri-nuclear ER of cardiomyocytes (Cheng et al., 2011). Alternatively, it is possible that GPER is localised close to the nuclear membrane for proximity to its downstream messengers that influence gene transcription (Fuentes and Silveryra, 2019). It is increasingly accepted that G protein-coupled receptors may be functional intracellularly (Gobeil et al., 2006), facilitated by GPER ligand oestrogens (and G-1) being membrane permeable (Prossnitz et al., 2007). This possibility of regional heterogeneity of GPER function within cardiomycytes requires further investigation to establish the complex signalling pathway and dynamic interactions of this receptor (Luo and Liu, 2020). GPER protein expression was found to increase in cardiomyocytes following OVx (Figure 1) which we posit may signify a protective role of GPER, upregulating in response to low oestrogen conditions. The upregulation of GPER in response to OVx may be specific to the heart as its expression decreased in the jejunum tissue of OVx mice, assessed using the same antibody as used this study (Samartzis et al., 2012). The change we observed in cardiomyocytes warranted further investigation into the effect of activating GPER in low oestrogen conditions.

The oestrogen receptors are studied separately in this study, with a focus on GPER due to its altered expression in response to oestrogen withdrawal. However, increasing evidence suggests these receptors cross-talk, particularly GPER and ERɑ, and interact to synergistically or antagonistically regulate signalling (Filardo et al., 2007; Fuentes and Silveryra, 2019). Furthermore, these receptors may compensate physiologically in the absence of one another and therefore, changes reported following GPER knockout for example, may be masked by compensatory ERɑ signalling. For example, cardiomyocyte-specific GPER KO led to LV dilatation and elevated end-diastolic pressure in male, but not female mice, possibly due to the effects of endogenous oestrogens, ERɑ and ERβ activity in females (Wang et al., 2017). It is important we first study these receptors independently to establish their individual contribution—particularly through the use of selective targeting as in this study using GPER agonist G-1—but future work should give consideration to receptor cross-talk.

### 4.2 The anti-arrhythmogenic effects of GPER activation

The effects of GPER activation were more pronounced in OVx compared with sham, with often no effect in sham animals. This increased response in OVx is likely due to the upregulation of GPER expression as a result of oestrogen withdrawal. This finding is supported by a study with rats that found GPER expression was upregulated in hypertensive animals and G-1 mediated improvements to relaxation and pressure, with no effect in the control group (de Francesco et al., 2013). In addition, another study found a reduction in systolic blood pressure in OVx rats after a 2-week *in vivo* treatment with G-1 but no change in the male or female control groups (Lindsey et al., 2009). This highlights the functional benefits of targetting GPER to limit the adverse effects of oestrogen withdrawal.

We previously found that Ca^2+^ handling was disrupted in OVx compared with sham (Yang et al., 2017) and consequently, OVx animals had larger CaT amplitudes. We now show that activation of GPER reduced the amplitude of CaT in OVx but not sham cardiomyocytes (Figure 2), which we suggest represents the capacity of GPER to mediate a protective response in stress-induced conditions such as in cardiomyocytes from females with low circulating oestrogens. There is reduced SERCA activity in the OVx cardiomyocytes incubated with G-1 (Supplementary Figure S3) which likely contributes to the smaller CaT amplitudes described. Mechanisitically, we propose that G-1 may interact with the SERCA regulatory protein, phospholamban and/or influence its phosphorylation status. Another consideration is the role of stromal interacting molecule 1 (STIM1) which functions in excitable cardiomyocytes by binding to phospholamban to disinhibit SERCA and increase its activity (Zhao et al., 2015). G-1 was found to prevent the *oligomerisation* of STIM1 in endothelial cells, leading to the inhibition of store-operated Ca^2+^ entry (Terry et al., 2017)—similar to our observations in OVx cardiomyocytes.

In contrast to our findings, G-1 increased CaT amplitude (and SL shortening) in mouse apical cardiomyocytes (Machuki et al., 2019). These findings likely represent heterogeneities between species in steroidogenesis or cardiac Ca^2+^-fluxes and highlight the complexity of oestrogenic signalling. Furthermore, marked apex-base differences in the expression of key Ca^2+^-handling proteins (NCx and LTCC) have been reported previously, with regional heterogeneities in response to 17β-oestradiol in female rabbit cardiomyocytes (Yang et al., 2012; Papp et al., 2017) - which may be worth exploring in our models.

Our data show a rise in spontaneous Ca^2+^ spark and wave frequencies in OVx (Yang et al., 2017), indicative of altered RyR sensitivity and changes considered to be pro-arrhythmic (Györke and Györke, 1998). There is evidence of a potential cardioprotective modulation of RyR activity by GPER through fewer uncontrolled release events (Figure 4) which may otherwise disturb Ca^2+^ regulation. Similarly, G-1 eliminated spontaneous Ca^2+^ spike activity in rat aortic smooth muscle a7r5 cells (Holm et al., 2012).

A negative inotropic effect of G-1 on cardiomyocyte contractility was observed in both sham and OVx as fractional SR Ca^2+^ release (Supplementary Figure S3) and SL shortening (Figure 2) were reduced. The effect of GPER on cardiac myofilaments remains to be established but we propose G-1 may depress actomyosin MgATPase activity and decrease myofilament Ca^2+^ sensitivity to reduce myocardial contractility. Oestrogen-mediated changes to myofilament function have been reported previously as activation of ERα reduced myofilament sensitivity through decreased phosphorylation of troponin I in mouse hearts (Kulpa et al., 2012). A similar action has been previously reported in smooth muscle cells in which G-1 induced relaxation of rat carotid arteries (Broughton et al., 2010) and, following OVx, in thoracic aortae (Lindsey et al., 2009). The opposite effect was observed in the aortae of GPER-deficient mice (Meyer et al., 2012), suggesting an anti-hypertensive role of GPER signalling. At the whole heart level, perfusion with G-1 reduced infarct size and limited contractile dysfunction following ischaemia and reperfusion episodes in rats (Deschamps et al., 2010).

G-1 shortened the APD and reduced the frequency of EADs in OVx cells during β-AR stimulation with isoprenaline (Figure 5). The APD prolongation in low oestrogen state increases the risk of abnormal membrane depolarisations, as shown previously in our OVx model (Yang et al., 2017), by allowing more time for LTCC reactivation (Morotti et al., 2012). Therefore the potential of G-1 to limit these membrane changes by shifting the voltage-dependent inactivation of the LTCC (Figure 3), would reduce the probability of Ca^2+^ channel openings during the “window” range of voltages, shortening the AP plateau (and consequently, AP duration) and protect the cell from abnormal depolarisations that risk triggering pro-arrhythmic extra beats (Fearnley et al., 2011). To support this, G-1 has previously been shown to attenuate the β-AR response in cardiomyocytes by abolishing isoprenaline-induced increases in *I*_Ca,L_ (Whitcomb et al., 2020) suggesting an important regulatory role of GPER in adrenergic signalling in the heart. The shortening of the AP in response to G-1 may also signify an interaction between GPER and the repolarising currents such as *I*_Kr_ but these remain to be investigated.

We also tested the effect of GPER activation on dofetilide-induced EAD formation and found G-1 abolished EADs in both sham and OVx cardiomoycytes (Figure 5). Dofetilide induces blockade of the *I*_Kr_ and is utilised medically for arrhythmia management. The adverse risk of uncontrolled dosing of dofetilide is EAD formation and *torsades de pointes* (Jaiswal and Goldbarg, 2014)*.* We suggest from our OVx model, that menopausal women receiving dofetilide treatment may experience more EADs and harmful arrhythmias compared with pre-menopausal women that have higher levels of circulating oestrogens. It was also observed in patients with congestive heart failure receiving dofetilide treatment that women had an increased risk of *torsades de pointes* (Ganjehei et al., 2011)*,* however women of all ages above 18 years (and therefore of varying menopausal statuses) participated in this study and the role of sex hormones in adverse dofetilide response is not assessed. In our study, we show that pre-treatment of cardiomyocytes with G-1 to activate GPER abolished EADs induced by dofetilide in both sham and OVx. Therefore, although further investigation is required, we suggest that GPER activation may reduce arrhythmogenic side effects and improve outcomes of dofetilide treatment in pre- and post-menopausal women. We conclude that GPER activation prevented the occurrence of pro-arrhythmic electrical activity, including vulnerability to abnormal depolarisations and suggest a protective role of the receptor.

### 4.3 Downstream signalling pathways of GPER

Acute activation of GPER mediates rapid changes including mobilising Ca^2+^ signalling within cardiomyocytes. GPER signals *via* the G-proteins, G_as_ and G_ai_, to rapidly activate a variety of downstream secondary messengers including pathways that involve ERK (Bopassa et al., 2010), PI3K (Deschamps et al., 2010) and cAMP signalling (Luo and Liu, 2020). This signalling has the capacity to modulate cellular activity by phosphorylating downstream targets such as ion channels, regulatory proteins and transcription factors (Fuentes and Silveryra, 2019). The conditions under which GPER signals *via* G_as_ or G_ai_ are yet to be determined. It is possible that, similar to β_2_-AR, GPER pivots to G_ai_ in response to stress conditions (Machuki et al., 2018). This pathway has been previously studied and G-1 stimulated an increase in the expression of AKT (Kang et al., 2012) and eNOS (de Francesco et al., 2013; Meyer et al., 2014) proteins. We propose such signalling *via* G_ai_ may underlie the G-1 response and Ca^2+^ changes observed following oestrogen withdrawal in this study.

### 4.4 Study limitations

Ovariectomy is widely used to model the effects of menopause but many peripheral tissues are capable of converting adrenally-released androgens into oestrogen because of the availability of aromatase. Hence plasma oestrogen concentrations are not necessarily indicative of localised organ levels. Reduction in ovarian hormones is acute and will not accurately represent natural menopause.

### 4.5 Clinical perspectives

Here we show, for the first time, that GPER activation induced electrophysiological changes and modulated Ca^2+^ signalling in cardiomyocytes in ways considered to be anti-arrhythmic. This suggests a potential role of GPER in modulating function and counteracting some types of arrhythmia particularly in post-menopausal women. The use of G-1 has great translational potential as it is currently a prototype drug in the US for treatment of obesity and metabolic syndrome, a disorder commonly arising post-menopausally (Sharma and Prossnitz, 2021).

## Data Availability

The raw data supporting the conclusions of this article will be made available by the authors, without undue reservation.
