# Supplementary figures and images for: GPER limits adverse changes to Ca2+ signalling and arrhythmogenic activity in ovariectomised guinea pig cardiomyocytes
Source: Front Physiol. 2022 Nov 10;13:1023755. doi: 10.3389/fphys.2022.1023755 (PMC9686394; doi:10.3389/fphys.2022.1023755)

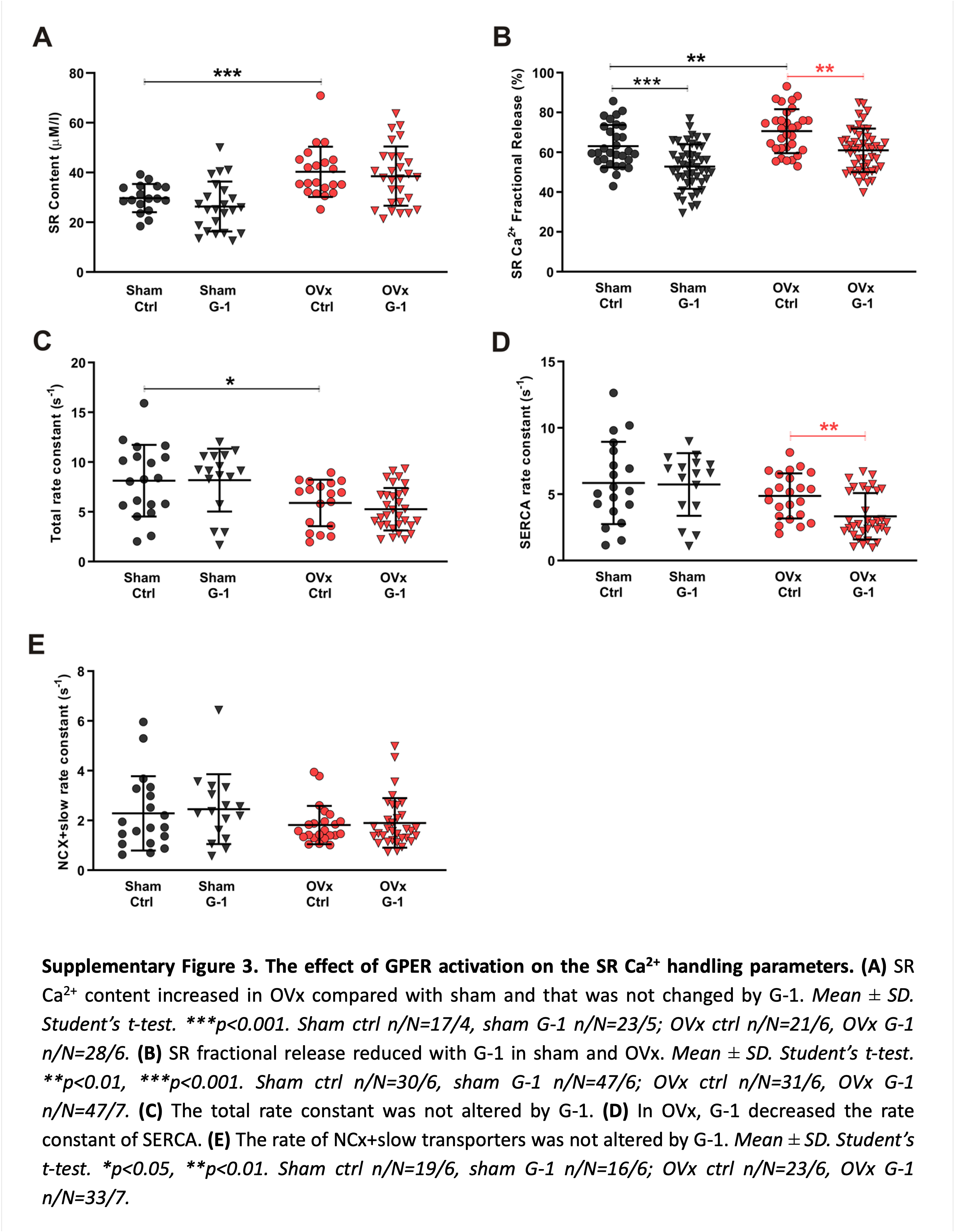

Supplement: Supplementary file 1 [file Presentation1.zip › Supplementary Figures/Supplementary Figure 3.tiff]

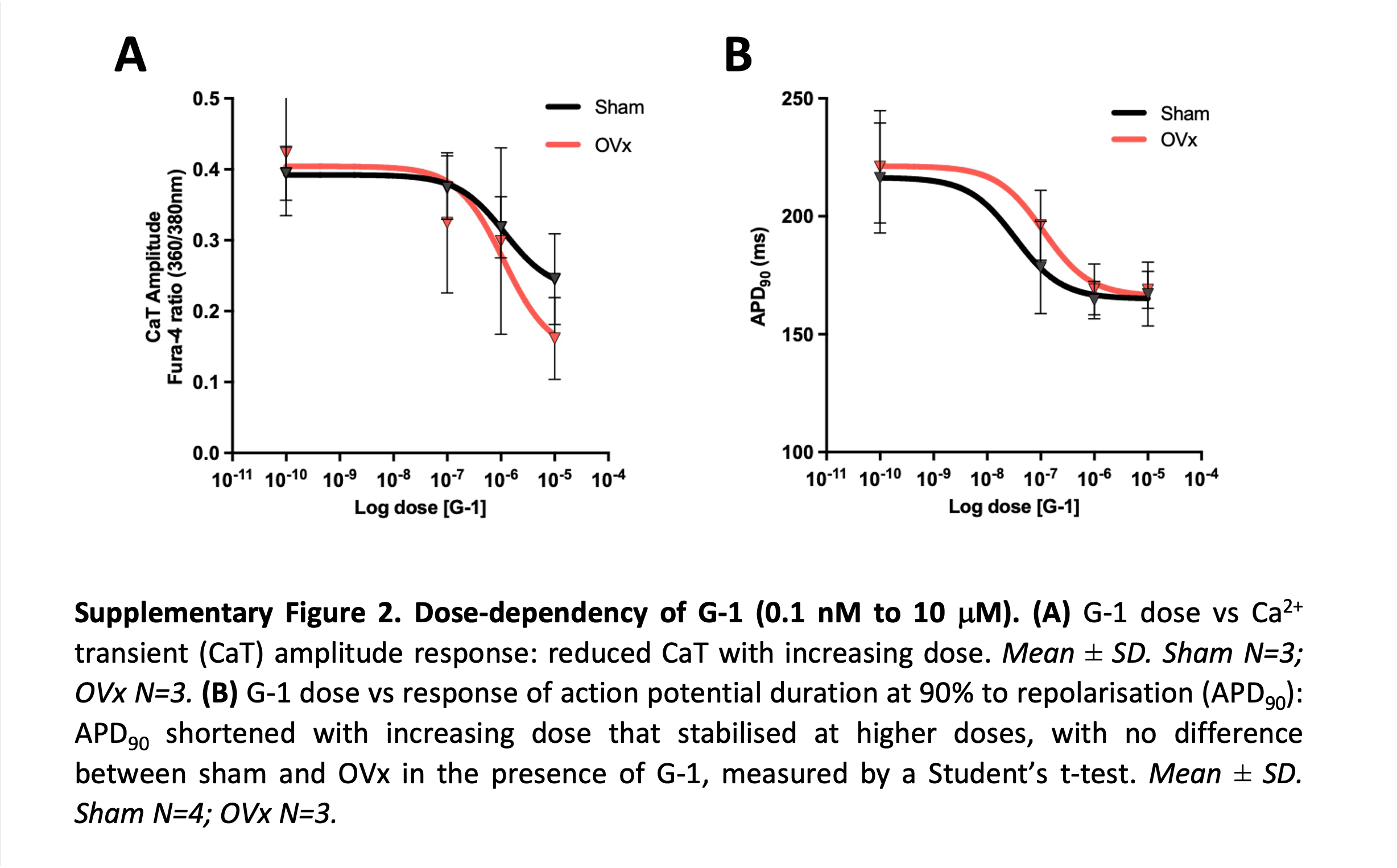

Supplement: Supplementary file 1 [file Presentation1.zip › Supplementary Figures/Supplementary Figure 2.tiff]

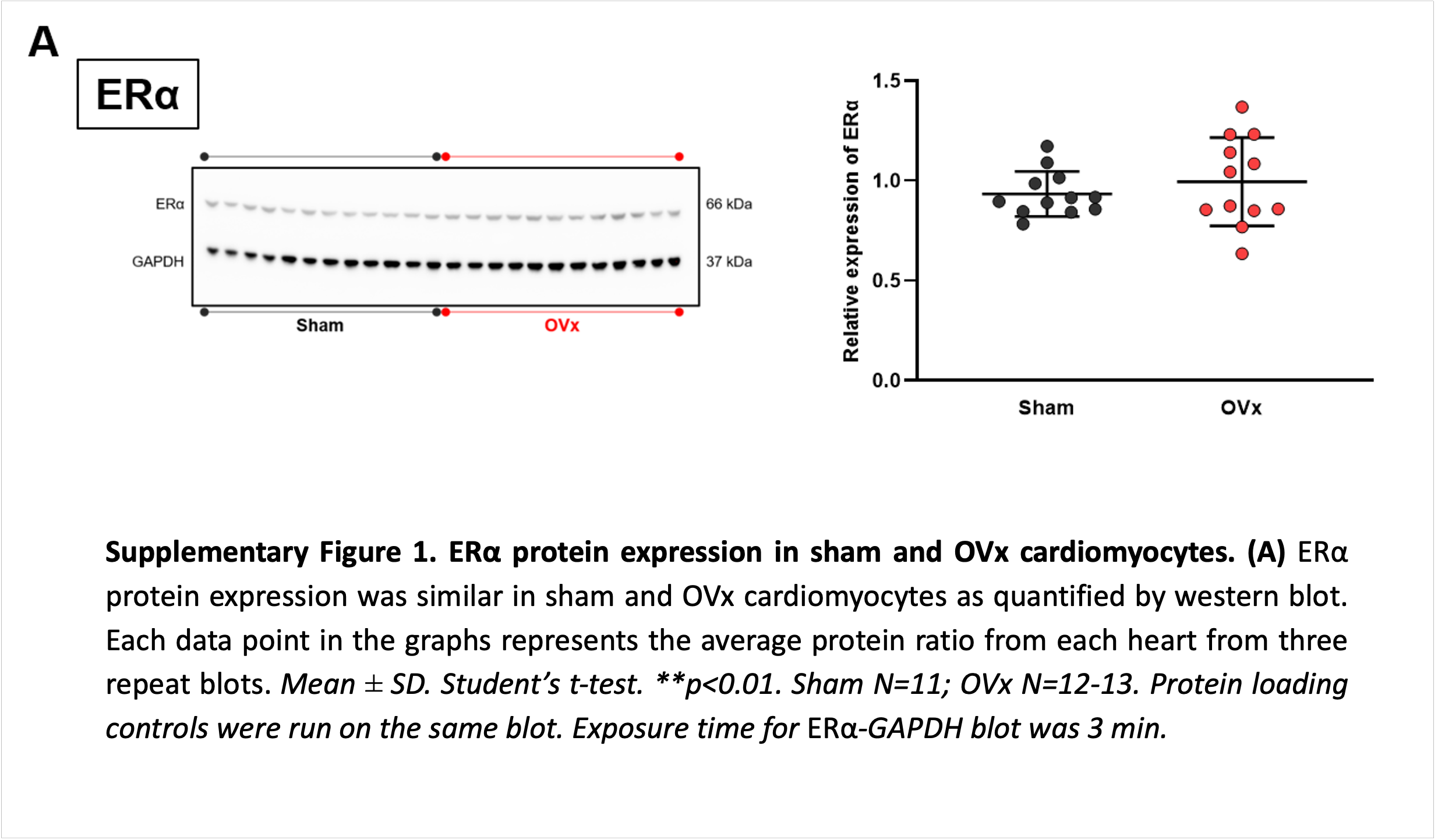

Supplement: Supplementary file 1 [file Presentation1.zip › Supplementary Figures/Supplementary Figure 1.tiff]
